# Nonneoplastic Renal Parenchymal Changes in Renal Cell Carcinoma With Tumor Thrombus

**DOI:** 10.7759/cureus.16531

**Published:** 2021-07-21

**Authors:** Ahmed Farag, Jeffrey J Gaynor, Felipe D Gaviria, Phillip Ruiz, Gaetano Ciancio

**Affiliations:** 1 Surgery, University of Miami Leonard M. Miller School of Medicine, Miami, USA; 2 Surgery, Zagazig University School of Medicine, Zagazig, EGY; 3 Surgery, The University of Miami Miller School of Medicine, Miami, USA

**Keywords:** non-neoplastic, renal cell carcinoma, tumor thrombus, global glomerulosclerosis, interstitial fibrosis

## Abstract

Introduction: Renal cell carcinoma may extend into the inferior vena cava (IVC) by the tumour thrombus (TT). Renal cell carcinoma with tumour thrombus (RCC/TT) could be associated with multiple collaterals making the surgery in cases of venous involvement very complex and challenging. The pathologic findings of non-neoplastic parenchymal changes in radical nephrectomy specimens of RCC/TT have not been well described.

Methods: We conducted a retrospective study of 200 nephrectomies for RCC/TT during eight years. We only included 22 patients who had a full histopathological examination of the resected nephrectomies, including the non-neoplastic parenchymal tissues.

Results: Median tumour thrombus level was III (range: II-IV), and median tumour diameter was 9.3 (range: 4-17) cm. Clear cell RCC was the most common tumour diagnosis in this cohort. Non-neoplastic renal pathologies included: (1) Global Glomerulosclerosis (GGS) in 90.9% (1-9% GGS in 15, 10-30% GGS in 4, >30% GGS in 1); (2) Interstitial fibrosis in 90.9% (mild in nine, moderate in nine, severe in 2); (3) Acute tubular injury in 14 (63.6%) patients; (4) Chronic inflammation in 77.3% (5-25% in 10, 26-50% in 7); (5) Arteriolosclerosis in all patients (mild, moderate and severe in 12, 9 and 1 patients, respectively); (6) Arteriolosclerosis: as none in 12, mild in six, moderate in four patients; (7) Focal Segmental Glomerulosclerosis in one patient. Our findings suggest that non-neoplastic parenchymal changes occur in the presence of RCC/TT. Neither tumour extension (via T-stage) nor tumour thrombus level were associated with the degree of any of these non-neoplastic parenchymal changes.

Conclusions: Knowledge of the existence of these non-neoplastic parenchymal changes in addition to determining the tumour margin(s) will be important in caring for and early determining whether any specific medical intervention(s) to help preserve renal function in the remaining contralateral kidney becomes warranted.

## Introduction

Renal cell carcinoma (RCC) is the most common malignant tumour of the kidney. Over 73,750 new cases were diagnosed in 2020 [1]. RCC has a myriad of presentations, and although infrequently encountered, it may extend by a tumour thrombus into the inferior vena cava (IVC) [2,3]. Chronic obstruction of the IVC by the tumour thrombus (TT) may occur. Such obstruction is often associated with the development of multiple collaterals, making the surgery in cases of venous involvement very complex and challenging [2-5].

The TT, which originates in the renal parenchyma due to its venous tropism, may extend into the renal vein, the IVC, and ultimately reach the right atrium. Once the TT reaches the IVC, the renal vein will be completely obstructed by the tumour thrombus [6,7]. Chronic obstruction of the renal vein could instigate pathologic non-neoplastic changes in the renal parenchyma. Initial interest in studying the non-neoplastic changes seen in nephrectomies performed for RCC began in 2006 by Bijol et al. [8]. Since then, multiple studies demonstrated a significant correlation between the non-neoplastic pathological findings and postoperative renal function in the contralateral kidney [9,10]. However, non-neoplastic parenchymal changes occurring in RCC/TT have not previously reported. Herein, we report the non-neoplastic pathologic findings in the renal parenchyma of patients with RCC complicated by TT.

## Materials and methods

Patients

After obtaining Institutional Review Board approval and following the ethical principles (as revised in 2013) of the Helsinki Declaration, a retrospective chart review was performed on patients who consecutively underwent surgical treatment for RCC/TT between September 2012 and June 2020 at our institution. During these eight years, 200 patients at our institution underwent surgical treatment of RCC/TT. We included 22 patients who had a full histopathological examination of the resected nephrectomies to include the non-neoplastic parenchymal tissues. We reported patients’ demographics, history of major comorbidities, history of smoking, preoperative and postoperative serum creatinine, intraoperative estimated blood loss, number of intra-operative units of blood transfusions (packed red blood cells) that were performed, and whether or not cardiopulmonary bypass was required during surgery. The cranial extent of the TT into the IVC was defined as Level I, II, III, or IV by a previous classification [6]. We analyzed the non-neoplastic pathologic changes in the nephrectomies’ parenchyma. The tissue analyzed as distal (more than 5mm from the tumour) to avoid the local effects of the tumour on adjacent tissue [11-13]. An evaluation and review of non-neoplastic renal parenchyma removed in the nephrectomies were performed to determine the level of injury present. A semiquantitative scoring/grading system was established, including: Global Glomerulosclerosis (GGS) and Focal Segmental Glomerulosclerosis (FSGS) score (0= 0%, 1= 1-9%, 2= 10-30%, 3= >30%); interstitial fibrosis (FIBR) score (percent involvement of interstitium - absent or 0 = 0-4%, mild or 1 = 5-25%, moderate or 2 = 26-50%, Severe or 3 = >50%); chronic inflammation (CI) score (percent involvement of interstitium; 0 = <5%, 1 = 5-25%, 2 = 26-50%, 3 = >50%); Arteriosclerosis (ASCL) and arteriolosclerosis (AASCL) score (percent luminal narrowing (mild, moderate and severe); Acute tubular injury (ATI) score: (0= none, 1= 1-10%, 2= 11-25%, 3= 26-45%, 4= 46-75%, 5= >75%).

Statistics

Descriptive statistical analysis was performed using SPSS 26 (Statistical Package for the Social Sciences), whereby distributions of categorical variables were reported in the form of percentages, and distributions of continuous variables were reported as medians along with corresponding ranges (as measures of dispersion), respectively. Tests of associations of the TT level and degree of tumour extension (via T-stage) with each non-neoplastic parenchymal changes were performed using Pearson (uncorrected) chi-squared tests, using a type I error of 0.05.

## Results

Patient Demographics

Over eight years between 2012 and 2020, 22 patients who had a full histopathological examination of the resected nephrectomies to involve the non-neoplastic parenchymal tissues were included in the study. The median age at the time of resection was 57 years (range: 33-79 years). There were five females (22.7%). The comorbidity prevalence was 77.3%, including hypertension in 15 patients (68.2%), diabetes mellitus type II in eight patients (36.4%), coronary artery disease in four patients (18.2%) and smoking history in six patients (27.3%). Table 1 shows the distributions of selected demographic and tumour characteristics.

**Table 1:** Demographics and tumor characteristics RCC- Renal cell carcinoma; IVC- Inferior vena cava

Variable	Patients (n=22)
Female, n (%)	5( 22.7%)
Age, median (y)	57 (range: 33-79)
Side of lesion, n (%)	
Left	12 (54.5%)
Right	10 (45.5%)
IVC involvement, n (%) Level I	8 (36.6%)
Level II	2 (9.1%)
Level III	9 (40.9%)
Level IV	3 (13.6%)
RCC type	
Clear cell type	17 (77.3%)
Papillary type	1 (4.6%)
Sarcomatoid type	1 (4.6%)
Unspecified	3 (13.6%%)
Tumor Grade	
Grade I	0
Grade II	4 (18.2%)
Grade III	9 (40.9%)
Grade IV	9 (40.9%)
T-Stage (n)	
T3a	7 (31.8)
T3b	5 (22.7)
T3c	8 (36.4)
T4	2 (9.1)
Tumor diameter, median (cm)	9.3 (range:4-17)

The median TT level was III (range: II-IV). The median tumour diameter was 9.3 cm (range, 4-17 cm); all tumours were ≥ 7 cm in diameter except for one patient in which it was 4 cm. T-stage T3a, T3b, T3c and T4 were seen in 7, 5, 8, and 2 patients, respectively, as per American Joint Committee on Cancer (AJCC) 2019 TNM staging system.7 All patients experienced some level of TT involvement. The most frequently encountered was level III with nine patients (40.9%), followed by level I in 8 patients (36.6%) and level IV in 3 patients (13.6%). Level II had the least amount in 2 patients (9.1%).

Pathological Examination

Histological examination revealed RCC clear cell type in 17 patients (77.3%) and papillary type in one patient (4.6%), sarcomatoid type in one patient (4.6%), and unspecified in 3 patients (13.6%). Global Glomerulosclerosis was present in all but two patients (90.9% of patients). Fifteen patients (68.2%) had GGS of 1-9%, 4 patients (18.2%) had 10-30% GGS, and 1 patient (4.6%) had >30% involvement. Regarding FIBR, it was present in 90.9% of specimens, with nine patients (40.9%) having mild (5-25%) involvement, nine patients having moderate (26-50%) involvement, and two patients having severe (>50%) involvement. ATI was absent in 8 patients (36.4%), while 5 patients (22.7%) had 1-10% ATI, 3 patients (13.6%) had 11-25% ATI, and 6 patients (27.3%) had 26-45% ATI. CI was found in 17 (77.3%) patients; in 10 patients (45.5%), there was 5-25% inflammation of interstitium, and in 7 patients (31.8%), there was 26-50% compromise. ASCL was present in all patients being mild, moderate and severe in 12 patients (54.5%), nine patients (40.9%) and one patient (4.6%), respectively. AASCL was present in 10 patients (45.5%) as mild/moderate, 6 patients (27.3%) as mild and 4 patients (18.2%) as moderate. FSGS was present in 1 patient. Casts were present in 6 patients (27.3%). Table 2 shows distributions of the non-neoplastic parenchymal characteristics.

**Table 2:** Pathologic changes observed in the non-neoplastic parenchyma of radical nephrectomy specimens with tumor thrombus. GGS: Global Glomerulosclerosis; ATI: Acute Tubular Injury; FIBR: Fibrosis; CI: Chronic Inflammation; ASCL: Arteriosclerosis; AASCL: Arteriolosclerosis.

Pathological outcome	N(%)
GSS score	
0 (none)	2 (9.1)
1 (<10%)	15 (68.2)
2 (10-30%)	4 (18.2)
3 (>30%)	1 (4.6)
ATI score	
0 (none)	8 (36.4)
1 (1-10%)	5 (22.7)
2 (11-25%)	3 (13.6)
3 (26-45%)	6 (27.3)
4 (46-75%)	0
5 (>75%)	0
FIBR score	
0 (none)	2 (9.1)
1 (mild)	9 (40.9)
2 (moderate)	9 (40.9)
3 (severe)	2 (9.1)
CI score	
0 (<5%)	5 (22.7)
1 (5-25%)	10 (45.5)
2 (26-50%)	7 (31.8)
3 (>50%)	0
ASCL score	
None	0
Mild	12 (54.6)
Moderate	9 (40.9)
Severe	1 (4.6)
AASCL score	
None	12 (54.6)
Mild	6 (27.3)
Moderate	4 (18.2)
Severe	0

Preoperative creatinine had a median of 1.0 (range: 0.47-1.8) mg/dl, and immediate postoperative creatinine median was 1.25mg/dl (range; 0.73-2.26mg/dl). Overall median EBL for all 22 patients was 675mL (range: 50 - 5000mL). Intra-operative blood transfusion was required in 11 patients; the median transfusion requirement was five units of packed red blood cells (range: 1-18 units). One patient required cardiopulmonary bypass during the surgery. EBL for the patient who required cardiopulmonary bypass was 850mL (n=1). An analysis of the 22 patients found no significant statistical associations of TT level and degree of tumour extension (via T-stage) with any of the non-neoplastic parenchymal changes (p>.10).

## Discussion

The main purpose of this study was to investigate the non-neoplastic parenchymal changes seen in 22 nephrectomy specimens of patients having RCC with TT and to determine whether the presence of the TT, which is known to cause venous congestion, may be associated with a greater likelihood of occurrence of non-neoplastic parenchymal changes. We analyzed the demographic and pathological findings of these 22 patients who received a radical nephrectomy at our institution over eight years, between 2012 and 2020.

We reported the parenchymal changes that were observed more than 5mm away from the tumour. This approach was taken in order to avoid reporting histological changes in the peritumoral parenchyma (≤5mm from tumor edge) in RCC cases which may be related to the mass compression effect of the tumor on surrounding parenchyma, possibly also resulting in a thickening of the small arterioles, narrowing and occlusion of the lumen. A mass compression effect could result in long-term ischemia and inflammatory responses with cellular infiltration that lead to these histological changes seen in peritumoral parenchyma [10,13].

When reviewing the literature, we did not find studies that evaluated non-neoplastic changes in cases of RCC with TT. Additionally, in those studies of RCC without TT reported, it was not specified whether TT was present with any of the tumours (we assumed their absence). Tumour size was noticeably more significant in our patient population when compared with other studies of RCC without TT [14]. All tumours except for one were ≥7 cm, and the median tumour size was 9.3cm. Clear cell RCC subtype was the most prevalent subtype in our study, which is a repeated finding of prior studies of RCC without TT. However, the proportion having the clear cell subtype was somewhat higher in our study, at 77.3% compared to 61% in the study of Noroozinia et al. [14].

Non-neoplastic pathologic changes were found in all of our patient population. Bijol et al. (2004) found that 38.2% of samples had normal non-neoplastic parenchyma, whereas, in our study, all of the samples had some parenchymal injury (GGS, FIBR or ATI) [8]. Another study performed by Noroozinia et al. of 85 patients with RCC demonstrated that 29.3% of patients had normal non-neoplastic parenchyma [14]. This rather significant difference may suggest a relationship between the presence of TT and the appearance of such non-neoplastic changes. However, it is interesting to note that the parenchymal changes did not appear to worsen with higher TT levels (III & IV) or higher T-stage in our study, suggesting that these changes could be associated with the mere presence of TT (i.e., obstruction of the renal vein) regardless of its cranial level.

FIBR was present in 90.9%, whereas, in a study by Garcia-Roig et al., FIBR was present in 17.8% of partial nephrectomies for RCC without TT [11]. GGS, ASCL and chronic inflammation were significantly more prevalent among our patients (90.9% vs 18%, 100% vs 16.8% and 77.3% vs 12.4%, respectively). However, when we evaluated the incidence of moderate to severe significant pathologic findings (such as glomerular sclerosis, interstitial fibrosis and vascular sclerosis), they were present in 72.7% of patients, which was similar to 69.3% of cases seen in RCC without TT [16].

Our study has some limitations. It is a single-centre experience with a small number of patients. A larger study that compares non-neoplastic parenchymal changes in RCC with and without TT would be more able to confirm whether or not a higher incidence of non-neoplastic pathological changes is caused by the presence of tumour thrombus. Furthermore, longer follow-up of these patients is needed to correlate the initial non-neoplastic parenchymal findings from the nephrectomy tissue with any chronic changes observed in renal function of the remaining contralateral kidney over time. While we believe that our sample of 22 patients having complete histopathological information represents a representative sample of the whole cohort of 200 patients with RCC/TT who received a radical nephrectomy at our institution during 2012-2020, we still cannot rule out the possibility that some type of selection bias existed for these 22 cases. Finally, to our knowledge, this is the first study to address non-neoplastic parenchymal changes seen in patients with RCC/TT, which should help the clinician to become more aware of the likely presence of subclinical renal disease and in potentially designing some type of medical intervention to protect the remaining contralateral kidney.

## Conclusions

We performed a retrospective chart review on patients who underwent surgical treatment for RCC/TT. We analyzed the non-neoplastic pathologic changes in nephrectomies’ parenchyma more than 5 mm from the tumour edge to avoid the local effects of the tumour on adjacent tissue. RCC/TT demonstrated non-neoplastic parenchymal changes, which were noticeably more prevalent than in RCC without TT cases previously reported. These changes may tend to increase with advanced stages of RCC in which renal vein obstruction occurs. Moreover, knowledge of the existence of these pathological changes, in addition to determining the tumour margin(s), will be necessary for caring for and early determining whether any specific medical intervention to help preserve renal function in the remaining contralateral kidney becomes warranted.
